# Differential Immediate and Sustained Memory Enhancing Effects of Alpha7 Nicotinic Receptor Agonists and Allosteric Modulators in Rats

**DOI:** 10.1371/journal.pone.0027014

**Published:** 2011-11-09

**Authors:** Morten S. Thomsen, Mona El-Sayed, Jens D. Mikkelsen

**Affiliations:** 1 Neurobiology Research Unit, University Hospital Copenhagen, Copenhagen, Denmark; 2 Neurosearch A/S, Ballerup, Denmark; College of Medicine, National Cheng Kung University, Taiwan

## Abstract

The α7 nicotinic acetylcholine receptor (nAChR) is a potential target for the treatment of cognitive deficits in patients with schizophrenia, ADHD and Alzheimer's disease. Here we test the hypothesis that upregulation of α7 nAChR levels underlies the enhanced and sustained procognitive effect of repeated administration of α7 nAChR agonists. We further compare the effect of agonists to that of α7 nAChR positive allosteric modulators (PAMs), which do not induce upregulation of the α7 nAChR. Using the social discrimination test as a measure of short-term memory, we show that the α7 nAChR agonist A-582941 improves short-term memory immediately after repeated (7× daily), but not a single administration. The α7 nAChR PAMs PNU-120596 and AVL-3288 do not affect short-term memory immediately after a single or repeated administration. This demonstrates a fundamental difference in the behavioral effects of agonists and PAMs that may be relevant for clinical development. Importantly, A-582941 and AVL-3288 increase short-term memory 24 hrs after repeated, but not a single, administration, suggesting that repeated administration of both agonists and PAMs may produce sustained effects on cognitive performance. Subsequent [^125^I]-bungarotoxin autoradiography revealed no direct correlation between α7 nAChR levels in frontal cortical or hippocampal brain regions and short-term memory with either compound. Additionally, repeated treatment with A-582941 did not affect mRNA expression of RIC-3 or the lynx-like gene products lynx1, lynx2, PSCA, or Ly6H, which are known to affect nAChR function. In conclusion, both α7 nAChR agonists and PAMs exhibit sustained pro-cognitive effects after repeated administration, and altered levels of the α7 nAChR *per se*, or that of endogenous regulators of nAChR function, are likely not the major cause of this effect.

## Introduction

Agonists and positive allosteric modulators (PAMs) of the α7 nicotinic acetylcholine receptor (nAChR) are currently being developed to ameliorate cognitive deficits in diseases such as schizophrenia, ADHD and Alzheimer's disease [1], [2].

The α7 nAChR desensitizes rapidly in response to high agonist concentrations *in vitro* [3], [4], which initially led to concern regarding its applicability as a clinical drug target [5], [6]. However, there does not seem to be development of tolerance regarding the procognitive effects of α7 nAChR agonists in animal models (reviewed in [7]). Thus, repeated α7 nAChR agonist administration has been shown to improve auditory gating, Morris water maze learning, classical eyeblink conditioning, inhibitory avoidance and novel object recognition [8]–[13]. Importantly, repeated, but not acute, administration of the α7 nAChR agonist TC-5619 improves performance in the novel object recognition test [14], indicating an enhanced effect with repeated administration in this test. Furthermore, it has been shown that agonists of nAChRs can produce long-lasting cognitive effects that outlast the presence of the compounds in the body [15], [16]. Furthermore, [^3^H]-nicotine binding sites correlate with performance in the Morris water maze task several days after nicotine administration [17], suggesting that the prolonged effects were mediated by an increased number of nAChRs. Specifically for the α7 nAChR, it has been shown that the α7 nAChR agonist, AZD0328 enhances novel object recognition and increases [^125^I]-bungarotoxin (BTX) binding in mice 4–48 hours after administration [18]. In a related study AZD0328 enhanced performance in a delayed response task in monkeys with effects evident more than one month after administration of the compound [19]. Taken together, these studies suggest that increased receptor numbers may underlie the sustained cognitive effects of nAChR agonists, although a direct correlation between α7 nAChR levels and cognitive performance has not been investigated.

PAMs of the α7 nAChR increase the response to an agonist and are divided into two types depending on whether they also decrease desensitization of the receptor (type II) or not (type I) [20]. Compared to agonists, there is much less data regarding the cognitive effects of α7 nAChR PAMs in animals, and no published clinical data. However, documented effects include improvements of pre-pulse inhibition and auditory gating as well as short- and long-term memory [21]–[25], resembling the behavioral effects of the agonists. Since PAMs do not activate the receptor *per se*, but modulate the effects of endogenous transmitters, they may enable more subtle regulation of α7 nAChR responses compared to agonists [2], but on the other hand, a lack of activation by the PAMs alone may hamper their effectiveness in patients with decreased levels of endogenous activation. It is thus not clear whether agonists or PAMs are preferable for clinical use, or whether there are qualitative differences between these types of compounds *in vivo* in terms of repeated administration.

We have recently demonstrated a fundamental *in vivo* difference between agonists and PAMs in that acute or repeated administration of the former, but not the latter, increases [^125^I]-BTX binding sites in the rat brain, reflecting an increased number of α7 nAChRs [26]. Given that upregulation of the α7 nAChR might underlie the enhanced procognitive effect seen with repeated administration of α7 nAChR agonists, as well as the long-lasting cognitive effects of these compounds, it is pertinent to examine whether α7 nAChR PAMs exhibit similar properties, since they do not induce upregulation of the receptor.

Here we use a rat social discrimination test to examine whether the α7 nAChR agonist A-582941 or the α7 nAChR PAMs, AVL-3288 (type I) or PNU-120596 (type II), has procognitive effects after acute or repeated administration, respectively, and whether such effects are long-lasting. In addition, we relate the behavioral effect of the compounds to their effect on α7 nAChR binding sites in the brain as well as activation of intracellular signalling cascades.

## Results

### A-582941 and AVL-3288 enhance performance in the social discrimination test

**Figure 1:**
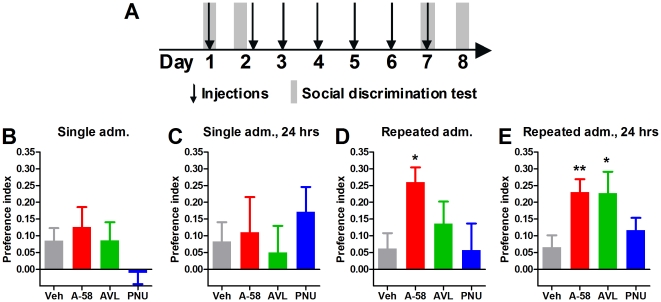
Effect of acute and repeated administration of α7 nAChR ligands in the social discrimination test. Rats were injected with A-582941 (A-58, 10 mg/kg), AVL-3288 (AVL, 1 mg/kg), PNU-120596 (PNU, 3 mg/kg), or vehicle (Veh, 5% DMSO, 8% Solutol in saline) once daily for seven days. (**A**) Diagram of the schedule for injections and behavioral testing. The rats were tested in the social discrimination test (**B**) on the day of the first injection (n = 13–20), (**C**) 24 hours after the first injection, but before the second injection (n = 10–14), (**D**) on the day of the seventh injection (n = 9–18), and (**E**) 24 hours after the last injection (n = 10–24). Neither compound affected social discrimination after a single injection. However, daily injection of A-58 for 7 days significantly increased the preference for exploring the novel juvenile compared to the familiar one, indicating an increase in short-term memory. Importantly, this effect was still detectable 24 hours after the last injection. At this time point, AVL also produced a significant increase in the preferatory index. * *P*<0.05 and ** *P*<0.01 indicate significant difference from vehicle in a one-way ANOVA with Dunnett's multiple comparison test. Error bars indicate the standard error of the mean.

Rats were injected subcutaneously (s.c.) with 10 mg/kg A-582941, 1 mg/kg AVL-3288, 3 mg/kg PNU-120596, or vehicle once daily for seven days. They were tested in the social discrimination test 4 times: on the day of the first injection; 24 hours after the first injection, but before the second injection; on the day of the seventh injection; and 24 hours after the last injection (Fig. 1A).

A single injection of either A-582941, AVL-3288, or PNU-120596 did not affect the preference of rats for a novel versus familiar juvenile, neither immediately (after a 2 hour intertrial interval) nor 24 hours after administration (Fig. 1B and C). However, when tested immediately after the last of seven daily injections, A-582941-treated rats exhibited a significantly enhanced preference index compared to vehicle-treated rats (Fig. 1D, *P*<0.05), whereas AVL-3288 and PNU-120596 had no effect. When tested 24 hours after the last administration, A-582941-treated rats still exhibited a significantly enhanced preference index (Fig. 1E, *P*<0.01). At this time point, AVL-3288-treated animals also has a significantly increased preference index (*P*<0.05), whereas there was no effect of PNU-120596.

The exploration time during the first trial of each test was analyzed, and there was no significant difference between groups in terms of exploration of the familiar stimulus rat (data not shown). Also, the total exploration time, i.e. the time spent exploring the familiar and novel stimulus rat, during the second trial did not significantly differ between groups in any of the tests.

### No correlation between performance in the social discrimination test and brain [^125^I]-bungarotoxin binding

After the final social discrimination test, rats were decapitated, and [^125^I]-BTX autoradiography was performed on coronal brain sections from the frontal cortex and hippocampus.

**Figure 2:**
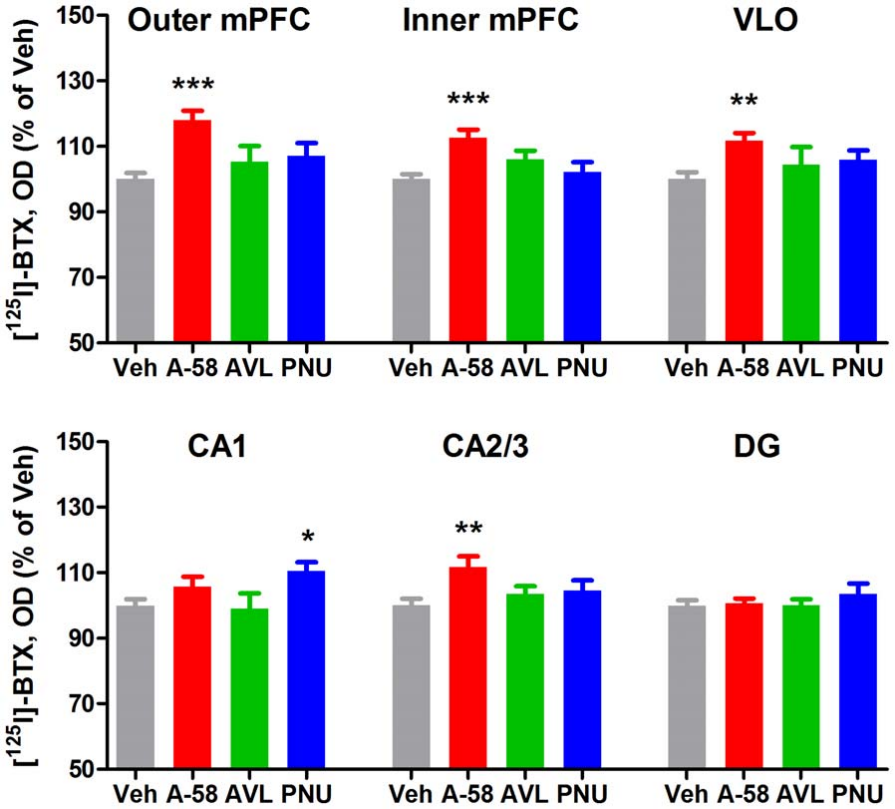
Brain [^125^I]-bungarotoxin binding in rats tested in the social discrimination test. Rats that underwent the injection and testing scheme described in Fig. 1 were decapitated immediately after the last social discrimination test. [^125^I]-bungarotoxin (BTX) binding was analyzed using optical density (OD) in the outer (I–IV) and inner (V–VI) layers of the medial prefrontal cortex (mPFC), the ventrolateral orbitofrontal cortex (VLO) as well as the CA1, CA2/3 and dentate gyrus (DG) regions of the hippocampus. A-582941 (A-58, n = 27)-treated rats had significantly increased [^125^I]-BTX binding in the outer and inner layers of the mPFC, the VLO and the CA2/3, compared with vehicle-treated rats (Veh, n = 26). PNU-120596 (PNU, n = 20) increased [^125^I]-BTX binding in the CA1, and AVL-3288 (AVL, n = 8) did not affect [^125^I]-BTX binding in any region measured. * *P*<0.05, ** *P*<0.01, and *** *P*<0.001 indicate significant difference from vehicle-treated rats in a one-way ANOVA with Dunnett's multiple comparison test performed separately for each region. Error bars indicate the standard error of the mean.

A-582941-treated animals had significantly increased [^125^I]-BTX binding in the outer and inner layers of the medial prefrontal cortex (mPFC), the ventrolateral orbitofrontal cortex (VLO), and the CA2/3 region of the hippocampus compared with vehicle-treated rats, whereas they did not differ from controls in the CA1 or dentate gyrus (DG) regions of the hippocampus (Fig. 2). PNU-120596 significantly increased [^125^I]-BTX binding in the CA1, whereas AVL-3288 did not affect binding in any region measured.

**Figure 3:**
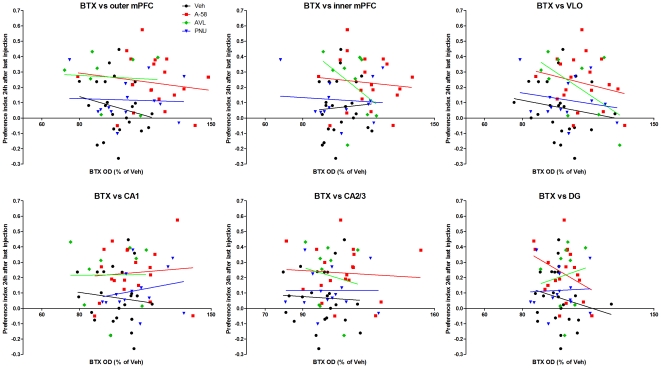
Correlation between performance in the social discrimination test and brain [^125^I]-bungarotoxin binding. The performance of rats in the social discrimination test 24 hours after the last of seven drug administrations, as described in Figure 1, was correlated with brain [^125^I]-bungarotoxin (BTX) binding, as described in Figure 2. Neither A-582941 (A-58, n = 19), PNU-120596 (PNU, n = 14), AVL-3288 (AVL, n = 8) or vehicle (Veh) treated rats displayed any significant correlations between performance in the social discrimination index and [^125^I]-BTX binding in any region measured. Correlation was determined as a slope that was significantly different from zero in a linear regression performed separately for each group in each region.

In an attempt to see whether there was a connection between α7 nAChR levels and cognitive performance, we correlated the performance of rats in the social discrimination test 24 hours after the last of seven drug administrations with brain [^125^I]-BTX binding. Neither A-582941-, PNU-120596-, AVL-3288- nor vehicle-treated rats displayed any significant correlations between performance in the social discrimination index and [^125^I]-BTX binding in any region measured (Fig. 3).

### PNU-120596 and AVL-3288 do not affect A-582941-induced immediate-early gene expression in the frontal cortex

**Figure 4:**
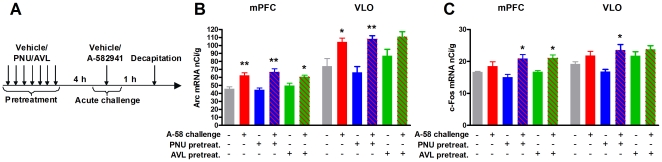
Repeated administration of PNU-120596 or AVL-3288 does not affect A-582941-induced Arc and c-Fos mRNA expression in the frontal cortex. Juvenile rats were administered PNU-120596 (PNU, 3 mg/kg), AVL-3288 (AVL, 1 mg/kg), or vehicle (5% DMSO, 8% Solutol in saline) once daily for 7 days. 4 hours after the last injection, the animals received an acute challenge of A-582941 (A-58, 10 mg/kg) or saline, and were decapitated 1 hour later. (**A**) A diagram of the experimental setup. Arc (**B**) and c-Fos (**C**) mRNA expression was analyzed using radioactive *in situ* hybridization in the medial prefrontal cortex (mPFC, layer II–VI) and the ventrolateral orbitofrontal cortex (VLO). A two-way ANOVA on Arc or c-Fos mRNA expression with repeated and acute treatment as the fixed factors showed a significant main effect of A-58-administration, but no interaction. Subsequent Bonferroni-corrected t-tests showed that the challenge with A-58 increased Arc mRNA in all groups except in the AVL-pretreated group in the VLO. Concerning c-Fos, there was only a significant effect of A-58 only in the PNU- and AVL-pretreated groups in the mPFC and only in the PNU-pretreated group in the VLO. N = 6. * *P*<0.05 and ** *P*<0.01 indicate significant difference from respective vehicle controls in a Bonferroni-corrected t-test performed separately for each region. Error bars indicate the standard error of the mean.

Juvenile rats were injected s.c. with 3 mg/kg PNU-120596, 1 mg/kg AVL-3288, or vehicle once daily for 7 days (pretreatment). 4 hours after the last injection, the animals received an acute challenge of 10 mg/kg A-582941 or vehicle and were decapitated 1 hour later (Fig. 4A). Radioactive *in situ* hybridization was performed for the immediate-early genes (IEGs) activity-regulated cytoskeleton-associated protein (*arc*) and *c-Fos*, on coronal brain sections from the frontal cortex.

A two-way ANOVA on Arc mRNA expression with pre-treatment and acute treatment as the fixed factors showed a significant main effect of acute administration of A-582941 in the mPFC and VLO (*P*<0.0001), but no main effect of the pre-treatment (*P* = 0.87 and *P* = 0.21, respectively) and no interaction between the effects of pre-treatment and acute treatment (*P* = 0.18 and *P* = 0.43, respectively, Fig. 4B). Subsequent Bonferroni-corrected t-tests showed that acute administration of A-582941 significantly increased Arc mRNA in the mPFC in vehicle-, PNU-120596-, and AVL-3288-pretreated rats, whereas the compound only increased Arc mRNA in the VLO in vehicle- and PNU-120596-pretreated rats.

A similar picture emerged for c-Fos mRNA, although the degree of induction with A-582941 was smaller overall (Fig. 4C). Thus, a two-way ANOVA on c-Fos mRNA expression showed a significant main effect of acute administration of A-582941 in the mPFC and VLO (*P*<0.0001 and *P* = 0.0006, respectively), but no main effect of the pre-treatment (*P* = 0.40 and *P* = 0.08, respectively) and no interaction (*P* = 0.13 and *P* = 0.12, respectively). Subsequent Bonferroni-corrected t-tests showed that acute administration of A-582941 significantly increased c-Fos mRNA in the mPFC in PNU120596- and AVL-3288-pretreated rats, and increased c-Fos mRNA in the VLO in PNU-120596-pretreated rats.

### Repeated A-582941 administration does not affect mRNA levels of endogenous regulators of α7 nAChR expression and function in the frontal cortex or hippocampus

Juvenile rats were administered 10 mg/kg A-582941 or vehicle s.c. once daily for 7 days and were decapitated 1, 4, 10, 24, 72 or 168 hours after the last administration. Vehicle-treated animals were decapitated after 1 hour (n = 6) or 7 days (n = 5), and since there was no difference in gene expression between the two groups (Student's t-test, data not shown), they were pooled. Tissue from the frontal cortex and hippocampus was dissected and analyzed using real-time qPCR.

**Figure 5:**
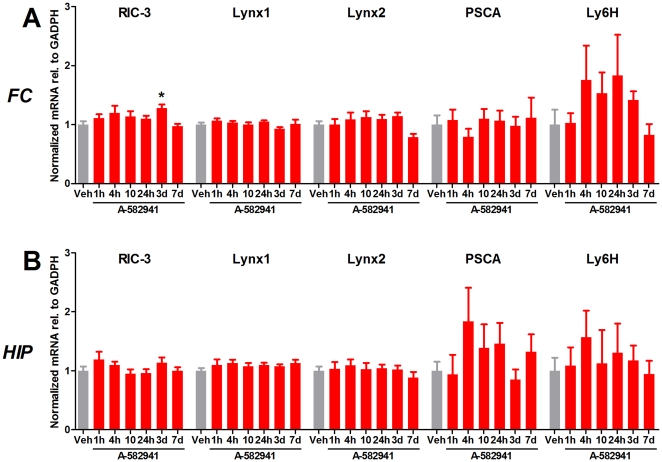
Effect of repeated A-582941 administration on expression of endogenous regulators of α7 nAChR function in the frontal cortex and hippocampus. Juvenile rats were administered A-582941 (10 mg/kg) or vehicle once daily for 7 days and were decapitated at the stated time after the last administration. Tissue from the frontal cortex (FC) and hippocampus (HIP) was dissected and analyzed using real-time qPCR. Vehicle-treated animals were decapitated after 1 hour (n = 6) or 7 days (n = 5), and since there was no difference between the two groups (Student's t-test), they were pooled. A-582941 significantly increased RIC-3 mRNA expression in the frontal cortex 3 days after the last administration. A-582941 did not significantly affect lynx1, lynx2, PSCA, or Ly6H expression in the frontal cortex or hippocampus at any time point. N = 6 (11 for vehicle). * *P*<0.05, ** *P*<0.01, and *** *P*<0.001 indicate significant difference from vehicle controls in a one-way ANOVA with Dunnett's multiple comparison test performed separately for each region. Error bars indicate the standard error of the mean.

A-582941 significantly increased RIC-3 mRNA expression in the frontal cortex 3 days after the last administration (Fig. 5). A-582941 did not significantly affect lynx1, lynx2, prostate stem cell antigen (PSCA), or Ly6H expression in the frontal cortex or hippocampus at any time point. However, there was a trend towards an increase in PSCA expression in the hippocampus and in Ly6H expression in both the frontal cortex and hippocampus.

## Discussion

The main findings in this study are that the memory-enhancing effect of the α7 nAChR agonist A-582941 in the social discrimination test is increased with repeated administration, and that repeated administration produces a sustained memory-enhancement. Furthermore, we demonstrate that the type I PAM, AVL-3288, also exhibits sustained memory-enhancing effects, whereas the type II PAM, PNU-120596, does not.

Repeated administration of A-582941 significantly enhanced short-term memory performance in the social discrimination test, whereas we did not observe an effect of a single administration of the compound. This finding is in accordance with a study showing that repeated, but not acute, administration of the α7 nAChR agonist TC-5619 improves performance in the novel object recognition test [14]. Similarly, 14 daily doses, but not one acute dose, of the α7 nAChR agonist SSR180711 mitigates a PCP-induced impairment in the novel object recognition test [27]. These results illustrate that analysis of the acute effects of α7 nAChR agonists does not reveal their full range of effect, and that the enhancement of effect is not restricted to the social domain. It should be noted that acute effects of A-582941 have been demonstrated in a social recognition test, similar to the test used here [11]. This difference may arise because we use higher doses than in the previous study used, or because our version of the test might be less sensitive to the effects of α7 nAChR agonists.

Neither single nor repeated administration of the α7 nAChR PAMs PNU-120596 nor AVL-3288 produced immediate effects on social discrimination. This demonstrates a fundamental difference between PAMs and agonists of the α7 nAChR in relation to the cognitive effects of repeated administration.

We further demonstrated that the pro-cognitive effect of repeated administration of A-582941 was sustained 24 hours after the last administration, whereas there was no effect 24 hours after a single administration. This suggests that neural changes occur with repeated administration that are not seen after a single exposure. Very low doses of the α7 nAChR agonist AZD0328 have been shown to enhance novel object recognition in mice 4–48 hours after a single administration [18]. Although exposure to ultra-low doses of α7 nAChR agonists have been speculated to confer particular effects [18], [19], this may indicate that our results are not due to a qualitative difference between the effects of a single and repeated administration, in terms of a prolonged effect, but rather an enhancement of a weak initial effect that is not picked up in the social discrimination test.

Despite the lack of an immediate effect, AVL-3288 enhanced short-term memory in the social discrimination test 24 hours after the last of seven injections. Contrarily, the type II PAM PNU-120596 had no long-term effect on social discrimination. This points towards a potential behavioral difference between type I and II α7 nAChR PAMs. Future studies are required to determine whether such long-term effects are unique to AVL-3288 or a general feature of type I α7 nAChR PAMs.

Several possible mechanisms may underlie the increased and sustained cognitive effects seen with repeated administration of A-582941 and AVL-3288, including a lowering of the threshold for LTP induction, increased nerve growth factor expression, IEG induction, and nAChR upregulation [15]. In line with this, we have previously demonstrated an increased amount of α7 nAChRs as well as an increased IEG response to a subsequent challenge with an α7 nAChR agonist after repeated administration with A-582941 in juvenile rats [26]. Contrarily, we show here that the IEG response to a challenge with an α7 nAChR agonist was not significantly altered in AVL-3288- or PNU-120596-pretreated animals. This lends credence to the idea that the immediate enhancement of the pro-cognitive effect of A-582941 with repeated treatment may be due to an enhanced responsiveness to α7 nAChR agonists at the cellular level, as reflected in an increased IEG response. This, however, does not explain the sustained effects on cognition seen with A-582941 and AVL-3288. The enhanced cellular responsiveness could be caused by an increase in α7 nAChR levels. An alternative explanation for the enhanced effect is that A-582941 may initially desensitize α7 nAChRs, thus obscuring an acute effect. This effect may be lessened with repeated administration, leading to the observed enhancement of short-term memory.

We further show that repeated administration of A-582941 increases BTX binding, as a measure of α7 nAChR levels, in adult rats. Contrarily, neither AVL-3288 nor PNU-120596 produced widespread increases in BTX binding, although PNU-120596 did significantly increase BTX binding in the CA1 region of the hippocampus. Importantly, there was no direct correlation between short-term memory 24 hours after the last of seven daily doses and BTX binding in the frontal cortex or hippocampus for any group of animals. Therefore, the sustained cognitive enhancement observed with A-582941 and AVL-3288, and previously demonstrated with other α7 nAChR agonists [16], [18], [19], is not likely due to an increase in receptor levels *per se*. There is also no correlation between short-term memory and BTX levels in vehicle-treated rats, suggesting that α7 nAChR levels are not the major determinant of performance in the social discrimination test.

It has been shown that α7 nAChR knockout mice display reduced attentional performance particularly with increased attentional load [28], [29], suggesting that the α7 nAChR is more important in situations of high attentional demand. It is therefore possible that a more demanding task might reveal a direct correlation between α7 nAChR levels and cognitive performance. Notably, [^125^I]-BTX autoradiography measures α7 nAChRs located both internally and at the cell surface. Therefore, we cannot exclude a possible correlation between surface receptors and performance. In addition, binding experiments, such as with BTX, favour conversion of receptors to a desensitized state, which has a higher affinity for ligand binding [30]. Therefore, BTX binding does not necessarily give an accurate measure of the pool of responsive receptors.

The lack of a widespread effect of AVL-3288 and PNU-120596 on BTX binding corresponds with our previous data showing a lack of effect of the PAMs PNU-120596 and NS1738 on BTX binding in non-performing juvenile rats [26], and suggests that the lack of effect of the PAMs is not due to a low cholinergic tone in non-performing animals, since the cholinergic tone would be expected to be much higher in animals that have undergone the social discrimination test [31].

A number of proteins have been described, which regulate the expression or function of nAChRs. These include RIC-3, which increases expression of α7 nAChRs [32], [33], and the lynx-like proteins lynx1, lynx2, PSCA, and Ly6H, which regulate the ion channel function of nAChRs, possibly by acting as allosteric modulators [34], [35]. We did not find a significant change in lynx1, lynx2, PSCA, or Ly6H mRNA expression in the frontal cortex or hippocampus of juvenile rats repeatedly administered A-582941, although there was a trend towards increased Ly6H expression in both the frontal cortex and hippocampus that matched the time-course of α7 nAChR upregulation, which has previously been assessed in the same animals [26]. However, Ly6H has been demonstrated not to bind to the α7 nAChR [36]. We did find a significant upregulation of RIC-3 mRNA in the frontal cortex 3 days after the final administration. However, the time course for upregulation of RIC-3 does not match that of α7 nAChR BTX binding sites, which peak after 4 hours [26]. It is therefore not likely that upregulation of RIC-3 is responsible for agonist-induced upregulation of the α7 nAChR

Taken together, these results suggest that the immediate procognitive effects of an α7 nAChR agonist are enhanced with repeated administration, possibly due to increased responsiveness at the cellular level, as reflected in an increased immediate-early gene response [26]. Furthermore, we demonstrate that both α7 nAChR agonists and type I PAMs may produce long-term improvement of cognition, and that this does not correlate with changes in α7 nAChR levels *per se* or that of known regulators of nAChR function.

## Materials and Methods

### Animals

A total of 154 Adult (172–300 g at the end of experiments), 84 juvenile (36 days, 100–140 g at the end of experiments) and 32 young juvenile (25–28 days, 52–88 g) male Wistar rats were purchased from Taconic Europe (Ll. Skensved, Denmark). The animals were acclimatized under standardized conditions with free access to food and water for a minimum of 5 days after arrival. Young juvenile rats were housed separately to avoid transfer of odors between animals. All experiments were conducted in accordance with the Declaration of Helsinki, the Danish National Guide for Care and Use of Laboratory animals and the European Communities Council Directive of 24 November 1986 (86/609/EEC).

### Drug treatment and tissue collection

A-582941 [11] and PNU-120596 [21] were synthesized at the Department of Medicinal Chemistry at NeuroSearch A/S. AVL-3288 (also known as XY4083 and CCMI) [22] was a kind gift from Kelvin W. Gee, University of California, Irvine. The compounds were injected s.c. at 2 ml/kg.

Four batches of adult rats (to a total of 154 rats) were tested in the social discrimination task. The rats were injected with vehicle (5% DMSO, 8% Solutol in 0.9% saline), 1 mg/kg AVL-3288, or 3 mg/kg PNU-120596 once daily for 7 days and tested on day 1, 2, 7, and 8. All animals that completed the fourth test (81 rats) were decapitated immediately after the test and their brains assayed for [^125^I]-BTX binding.

36 juvenile rats were injected with vehicle (5% DMSO, 8% Solutol in 0.9% saline), 1 mg/kg AVL-3288, or 3 mg/kg PNU-120596 once daily for 7 days, and 4 hours after the last injection, they received an injection of vehicle or 10 mg/kg A-582941 and were decapitated 1 hour later.

48 juvenile rats were injected with vehicle (0.9% NaCl) or 10 mg/kg A-582941 once daily for 7 days and decapitated 1, 4, 10, 24 hours, 3, or 7 days after the last injection.

For all experiments, the brains were dissected and divided into two hemispheres. One hemisphere was frozen directly in powdered dry ice, whereas the prefrontal cortex and hippocampus were dissected from the other hemisphere before freezing.

### Social discrimination

The social discrimination test was modified from [37]. Briefly, an adult male Wistar rat was put into a clean standard rat cage (43×27×18 cm) with a small amount of bedding ≥60 min before the test. Immediately before testing, the cage was gently moved to a testing platform fitted with a video camera, the lid was removed, and a young juvenile (25–28 days) male rat was introduced into the cage. A transparent plastic plate was quickly placed on top of the cage, and the rats were recorded for 5 min (trial 1). After trial 1, the juvenile rat was removed, the lid replaced, and the cage moved back to its original place. Drug injections, were given immediately after trial 1. After an inter-trial interval of 120 min, a second trial was performed. Trial 2 was identical to trial 1, except that two juvenile rats were introduced – the same juvenile as in trial 1 (familiar), and a juvenile that the adult rat had never seen before (novel). The amount of time the adult rat spent exploring the juveniles in trial 1 and 2 was measured by an observer blind to the treatment of the rats. Social exploration was defined as including sniffing, licking, and chewing fur of the juvenile, as well as pawing and close following of the juvenile. Exploration initiated by the juveniles was not included. The exploration in trial 2 is presented as a preference index, which is the difference between the time spent interacting with the novel and familiar juvenile divided by the total interaction time. The young adult rats were used for multiple tests, but were never used for more than one test with the same adult rat.

### Autoradiography

Brain hemispheres were cut into 12 µm serial coronal sections on a cryostat and directly thaw-mounted onto super frost glass slides. Sections were collected in parallel series with 4–6 sections per glass slide throughout the prefrontal cortex (6 series, 2.8–3.2 mm anterior to Bregma) and the dorsal hippocampal region (6 series, 3.6–4.0 mm posterior to Bregma) (Paxinos and Watson 1986). Two slides from each animal were thawed at room temperature for 30 min, followed by 30 min hydration in 50 mM Tris buffer, pH 7.3 (binding buffer). Slides were then incubated 2 hours in binding buffer containing 0.5 nM [^125^I]Tyr-54-mono-iodo-α-bungarotoxin (2,200 Ci/mmol, Perkin Elmer, Skovlunde, Denmark) to asses total binding. For analysis of non-specific binding, 1 mM (−)-nicotine (Sigma-Aldrich, Brøndby, Denmark) was included in the incubation. Slides were then briefly washed in binding buffer, followed by 2×30 min washes in ice-cold binding buffer and rinsed briefly in ice-cold distilled water. Slides were then dried under an air stream and fixated overnight at 4°C in a sealed chamber containing paraformaldehyde vapor. Finally, slides were dried 2–3 hours in an excicator, exposed to a BAS-MS2040 phosphor imaging plate (Science Imaging Scandinavia AB, Nacka, Sweden) for ∼4 hours and scanned with a BAS-2500 imaging plate scanner (Fujifilm Europe GmbH, Düsseldorf, Germany).

### Quantitative assessment of mRNA levels

Total RNA was isolated with Trizol Reagent (Sigma-Aldrich, Brøndby, Denmark) according to the manufacturer's directions. The samples were dissolved in RNase-free water and RNA content was quantified using a Nanodrop ND-1000 spectrophotometer (Nanodrop Technologies, Wilmington, DE). Extracted RNA was reverse transcribed into single-stranded cDNA with the ImProm-II™ reverse transcription kit (Promega, Madison, WI) according to the manufacturer's directions using oligo(dT)_15_ primers, 6 mM MgCl_2_, and 20 units of RNase inhibitor.

Real-time qPCR reactions were performed in a total volume of 20 µl, containing 1 µl sample cDNA, 1× Brilliant II SYBR green mastermix (Stratagene, La Jolla, CA), and 15 pmol each of the forward and reverse primer (DNA technology, Aarhus, Denmark). PCR was performed on a Light Cycler 480 (Roche, Indianapolis, IN) with a 10 minute preincubation at 94°C followed by 40 cycles of 30 seconds at 94°C, 45 seconds at 60°C and 1.5 minutes at 72°C. All primers were validated by using serially diluted cDNA to establish a standard curve, and by confirming the size of the product on a DNA gel (data not shown). The primers used are described in Table 1. Quantification of mRNA expression was performed according to the comparative C_T_ method as described in [38]. For each sample, the amount of target mRNA was normalized to that of the reference gene GAPDH.

**Table 1:** Primers used for real-time qPCR.

Gene	Forward primer 5′-3′	Reverse primer 5′-3′	Product size
RIC-3	CAGCACTGATAACACACATGTGG	GCAGGCTGCTTTCACTCAAAA	75 bp
Lynx1	ACCACTCGAACTTACTTCACC	ATCGTACACGGTCTCAAAGC	81 bp
Lynx2	GTTCTGGCTTCCAGGGCTGG	GGCTGCTGACGATGCACACG	191 bp
PSCA	GCCCTACCAGTTCTGATCAG	TCACACCCACCTAGCTTCAT	154 bp
Ly6H	CTACTGGCCTTGCTTCTCTG	AATGATCCTTCCTGCTGCTG	163 bp
GAPDH	CATCAAGAAGGTGGTGAAGCA	CTGTTGAAGTCACAGGAGACA	93 bp

### *In situ* hybridization

*In situ* hybridization was performed as previously described [26]. Two different synthetic oligonucleotide DNA probes (DNA Technology, Aarhus, Denmark) complimentary to bases 789–839 of rat Arc cDNA [39] and bases 132–179 of rat c-Fos cDNA [40], respectively, were used.

### Quantification and data analysis

Mean optical densities from autoradiography and *in situ* hybridization were quantified in the regions of interest using a computer image analysis system (Quantity One®, Bio-Rad, CA) by an observer blinded to the treatment of the animals. The individual value for each region was calculated as the average measurement from three individual sections. For autoradiography, the average value from an adjacent slice with non-specific binding was subtracted to yield specific binding. For *in situ* hybridization, background values were measured for each slide individually in a tissue-free area and subtracted from each measurement on that slide. The presented values are the measured value minus the background/non-specific measurement, normalized to the level of the respective vehicle group.

Data were analyzed using two-way ANOVA, one-way ANOVA followed by Dunnett's Multiple Comparison test, or Bonferroni-corrected t-tests as appropriate. Linear regression was used to correlate [^125^I]-BTX binding with performance in the social discrimination test. The statistical calculations were performed using GraphPad Prism version 5.03 for Windows (GraphPad Software, San Diego, USA). All data are presented as mean±standard error of the mean, and a *P*-value of less than 0.05 was considered statistically significant.
